# Proteins that bind methylated DNA and human cancer: reading the wrong words

**DOI:** 10.1038/sj.bjc.6604374

**Published:** 2008-05-27

**Authors:** L Lopez-Serra, M Esteller

**Affiliations:** 1 Cancer Epigenetics Group, Spanish National Cancer Research Centre (CNIO), Melchor Fernández Almagro 3, Madrid 28029, Spain; 2 Cancer Epigenetics and Biology Program (PEBC), Catalan Institute of Oncology (ICO), 08907 L'Hospitalet, Barcelona, Catalonia, Spain

**Keywords:** methyl-CpG-binding domain proteins, DNA methylation, epigenetics

## Abstract

DNA methylation and the machinery involved in epigenetic regulation are key elements in the maintenance of cellular homeostasis. Epigenetic mechanisms are involved in embryonic development and the establishment of tissue-specific expression, X-chromosome inactivation and imprinting patterns, and maintenance of chromosome stability. The balance between all the enzymes and factors involved in DNA methylation and its interpretation by different groups of nuclear factors is crucial for normal cell behaviour. In cancer and other diseases, misregulation of epigenetic marks is a common feature, also including DNA methylation and histone post-translational modifications. In this scenario, it is worth mentioning a family of proteins characterized by the presence of a methyl-CpG-binding domain (MBDs) that are involved in interpreting the information encoded by DNA methylation and the recruitment of the enzymes responsible for establishing a silenced state of the chromatin. The generation of novel aberrantly hypermethylated regions during cancer development and progression makes MBD proteins interesting targets for their biological and clinical implications.

## DNA METHYLATION AND ITS MEDIATORS

DNA methylation is one of the most important mechanisms for epigenetic silencing in mammals. It is a key element in heterochromatin formation and maintenance, and is involved in processes such as X-chromosome inactivation and gene imprinting. DNA methylation also occurs at repetitive sequences to ensure chromosome stability. DNA methylation is developed by a family of DNA methyltransferase enzymes (DNMTs) that add a methyl group to the fifth carbon position of cytosine when followed by a guanine nucleotide. Methylation of DNA is accompanied by histone post-translational modifications that modulate DNA function, regulating chromatin structure and determining the transcriptional state of the DNA wrapped around it.

The crosstalk between DNA methylation and histone modifications is established by different nuclear factors. Among them, it is particularly remarkable a family of proteins that contain a Methyl-CpG-binding domain commonly known as MBD proteins that recognises single methylated CpG dinucleotide (Hendrich and Bird, 1998). MBD family members recruit histone modifying and chromatin remodeling complexes to methylated sites. The MBD family of proteins is composed by five members namely MeCP2, MBD1, MBD2, MBD3 and MBD4. MeCP2 was the first characterised member and its 70 amino acids region corresponding to the MBD was used for database identification of the remaining MBD containing proteins. The binding affinity of MBD proteins differs between them, as was demonstrated that MBD2 is the MBD protein with higher methylated DNA-binding affinity *in vitro* (Fraga *et al*, 2003).

## DNA METHYLATION AND CANCER

In cancer, DNA methylation patterns are profoundly altered. While there is a global decrease of CpG methylation, promoter CpG island of tumour suppressor genes become hypermethylated. In this landscape, chromosome instability has been proposed to be associated with global genomic demethylation, whereas aberrant hypermethylation of promoter CpG island leads to gene inactivation. CpG islands are present at the promoter region of approximately 60% of human genes. These regulatory regions are commonly unmethylated under physiological conditions, however in cancer the promoter CpG island of many tumour suppressor genes become hypermethylated. Cellular pathways affected by CpG island hypermethylation include cell cycle, apoptosis, cell adherence, DNA repair, carcinogen metabolism and so on. For instance, genes such as p16^INK4a^, hMLH1 and BRCA1 are silenced in many types of cancer due to CpG island hypermethylation. The mechanisms that define which of these sequences become methylated is starting to be evident and it has been suggested that it is a combination of cellular selection of randomly methylated genes with targeted pathways.

CpG island hypermethylation of tumour suppressor genes is such a common fact in cancer that tumours can be classified according to their methylation profile (Esteller *et al*, 2001; Esteller 2008). For instance, BRCA1 CpG island hypermethylation occurs mainly in breast and ovarian cancer, and hMLH1 undergoes promoter hypermethylation specifically in colon, gastric and endometrial tumours profile (Esteller *et al*, 2001; Esteller 2008). The characteristic methylation profiles associated with each tumour type could be explained by selective advantage, the methylation profile of a cell in a particular tissue confers a selective advantage to the survival only in that environment.

It has also been reported that aberrant promoter hypermethylation patterns are also involved in miRNA silencing (Saito *et al*, 2006; Lujambio *et al*, 2007). miRNA are small non-coding RNAs that inhibit protein expression of target genes. In cancer, miRNA expression profiles differ between normal tissues and tumour samples and between different tumour types (Lu *et al*, 2005), and CpG island hypermethylation accounts for the transcriptional silencing of a subset of miRNAs (Saito *et al*, 2006; Lujambio *et al*, 2007).

In cancer, not only DNA methylation but also histone modifications are altered. Decreased levels of histone H4 lysine 16 monoacetylation and histone H4 lysine 20 trimethylation have been described as common hallmark in cancer (Fraga *et al*, 2005). Mutations in the machinery that are involved in epigenetic marks are also involved in tumorigenesis, as reported for HDAC2 (Ropero *et al*, 2006).

**Figure 1:**
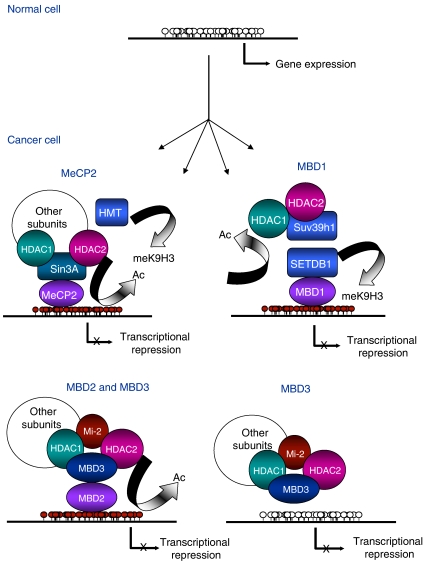
Mechanisms of epigenetic silencing by MBD proteins. Red and white circles represent methylated and unmethylated CpGs, respectively.

In this context, it was suggested that MBD proteins were bound to aberrantly methylated sequences, which trigger transcriptional misregulation (Figure 1 and Table 1). In cancer cells MBD protein levels are abnormal but this can be due to the increased cell proliferation in these cells. In B-cell chronic lymphocytic leukaemia, MeCP2 and MBD2 levels positively correlate with Histone Methyl Transferases levels (Kn *et al*, 2004) suggesting that epigenetic machinery is deregulated. In breast cancer MeCP2 is over expressed and appears to be associated with oestrogen receptor positivity (Muller *et al*, 2003). Polymorphisms in the sequence of MBD proteins have been reported and some of them seem to be associated with cancer risk, for instance, polymorphisms in MBD1 increase the overall risk of lung cancer (Jang *et al*, 2005) while polymorphisms in MBD2 is associated with reduced risk of breast cancer among premenopausal women (Zhu *et al*, 2005). However, mutations in the MBD of MeCP2, MBD2 and MBD3 have not been described in colon nor in endometrial cancer cell lines (Ropero *et al*, 2006).

MBD protein occupancy of hypermethylated promoters in tumour suppressor genes were reported in a large panel of human cancer cell lines and their binding correlates with gene silencing (Table 1). For instance, different MBD proteins were found occupying the hypermethylated promoter of p16^INK4a^ or DAPK1 and the type of MBD bound to each promoter seems to be tumour type and gene specific (Lopez-Serra *et al*, 2006). MBD proteins have also been found to be associated with the hypermethylated version of CpG islands embedded in miRNAs in colon cancer cells (Lujambio *et al*, 2007).

More recently, interference of MBD proteins, using hairpin RNA molecules (RNAi), were shown to release the transcriptional expression of sets of hypermethylated genes without altering their DNA methylation status (López-Serra *et al*, 2008). These results support the notion that MBD proteins are involved in establishing a crosstalk between DNA methylation and gene silencing machinery recruitment but not in the establishment of DNA methylation patterns (López-Serra *et al*, 2007). Some of the genes silenced by DNA hypermethylation and the subsequent binding of MBD proteins and chromatin reorganisation became expressed again when we carried out MBD depletion. The re-expression of these genes correlates with cell proliferation and tumorigenic properties decrease. Taking into account the fact that MBD proteins bind aberrantly hypermethylated promoters, they can also be used to track novel genes that become epigenetically silenced in cancer by combining chromatin immunoprecipitation assays with hybridization on genomic microarrays (Ballestar *et al*, 2003).

## MeCP2

MeCP2 is the founding member of the MBD family of proteins. It is a 50 kDa protein encoded by a gene located on the X-chromosome which is composed of four exons that lead to two different splicing variants called MeCP2α and MeCP2β depending on the presence or absence of the exon 2.

This protein contains the MBD in the N-terminal region and a transcriptional repression domain (TRD) near the C-terminal region. The TRD domain includes a nuclear localization signal.

Repression by MeCP2 is mediated by chromatin remodelling complexes recruitment to methylated DNA sequences (Figure 1). The TRD domain interacts with Sin3A, a complex containing histone desacetylase enzymes HDAC1 and HDAC2. Histone deacetylation is not the only way in which MeCP2 represses transcription and establishes heterochromatin formation, it is also known that MeCP2 interacts with a complex containing lysine 9 of histone H3 methyltransferase activity (Fuks *et al*, 2003) although its identity is not yet known.

Mutations of the MBD and TRD domains of MeCP2 lead to Rett syndrome, a neurodegenerative disease that affects mostly females. Rett syndrome is characterised by mental retardation, autism, microcephaly, breathing defects, characteristic hand movements and others symptoms that appear between 6–18 months of age after a normal initial development. MeCP2 knock out mice are viable but show Rett syndrome symptoms like motor coordination problems (Guy *et al*, 2001). It seems that MeCP2 plays an important role in neural differentiation regulation since its lack leads to this neurodegenerative disease. In cancer cells, inhibition of MECP2 expression stops the growth of cancer prostate cells, while its ectopic expression confers a growth advantage (Bernard *et al*, 2006).

## MBD1

MBD1 is a 55 kDa protein encoded by a gene located on chromosome 18. MBD1 contains the MBD in the N-terminal region, a TRD in the C-terminal region and 2 or 3 CxxCxxC domains located between the MBD and the TRD called CxxC1, CxxC2 and CxxC3. The number of CxxCxxC domains depends on alternative splicing events. Isoforms containing the CxxC3 are able to bind unmethylated DNA so these proteins repress transcription not only of methylated sequences but also of unmethylated regions (Fujita *et al*, 2000).

MBD1 repressive activity were reported to be mediated by lysine 9 (K9) of histone H3 methylation through SETDB1 histone methyl-transferase (HMT) recruitment (Klose and Bird, 2006) (Figure 1). MBD1 also interacts with Suv39h, another HMT that methylates K9 of histone H3 (Fujita *et al*, 2003).

Mice lacking MBD1 have no development problems and exhibit an apparently normal phenotype. However, pluripotency neuronal cells show decreased neuronal differentiation and chromosome instability (Klose and Bird, 2006).

## MBD2

Methyl-CpG-binding domain 2 is a protein encoded on chromosome 18. It has two splicing variants, MBD2a (43, 5 kDa) and MBD2b (29.1 kDa), that differ in the position of the translation start site.

Repressive activity of MBD2 is mediated by MeCP1, an ATP dependent chromatin remodelling complex formed by MBD2 and Mi-2/NuRD complex (Fatemi and Wade, 2006) (Figure 1). Methyl-CpG-binding domain 2 is also involved in methylation-dependent gene silencing of Xist, a gene that plays an essential role in X-chromosome inactivation in mammals. The product of this gene is a non-coding RNA that covers the X-chromosome that remains inactive. Methyl-CpG-binding domain 2 *knock out* mice do not present defects during embryonic development but MBD2 deficient females show abnormal maternal behaviour (Hendrich *et al*, 2001). However, Apc^(Min/+)^ mice (a mouse model for colon adenomatous polyps) lacking MBD2 show up to 10 times reduced intestinal tumorigenesis and more localised tumours than those Apc^(Min/+)^ MBD2^+/+^ (Sansom *et al*, 2003). Moreover, MBD2 level changes are associated with significant changes at certain cytokines levels involved in lymphocyte T maturation (Fatemi and Wade, 2006). A controversial role of MBD2 as a DNA demethylase has also been proposed (Bhattacharya *et al*, 1999).

MBD2 has also been related to GSTP1 DNA methylation-dependent silencing in hepatocellular carcinoma (Bakker *et al*, 2002). The CpG island of this gene becomes hypermethylated during the pathogenesis of human hepatocellular carcinoma and MBD2 appears bound to GSTP1 promoter region leading to gene silencing.

## MBD3

The gene coding MBD3 is located on chromosome 19. Methyl-CpG-binding domain 3 has two splicing variants that differ in size, 32 and 20 kDa. In mammals, MBD3 is unable to bind methylated DNA due to the presence of two alterations within the MBD. Despite the lack of a functional methyl-CpG-binding domain, MBD3 plays an important role in DNA methylation-dependent events. MBD3 deficient mice die early during embryonic development (Fatemi and Wade, 2006).

Methyl-CpG-binding domain 3 is an integral subunit of the chromatin remodelling complex Mi-2/NuRD (Figure 1). This complex is also composed by histone deacetylases HDAC1 and HDAC2, histone-binding proteins RbAp46 and RbAp48, the chromatin-remodelling protein Mi-2 and metastasis-associated proteins MTA1 and MTA2. Mi-2/NuRD association with MBD2 leads to MeCP1 complex, initially identified as a methylated DNA-binding protein and later like a complex containing MBD proteins.

Mi-2/NuRD-mediated silencing of methylated genes was hypothesised as starting with MBD2 binding to methylated DNA followed by Mi-2/NuRD recruitment. However, MBD2 and MBD3 associate to the Mi-2/NuRD complex in a mutually exclusive way with different biochemistry and functional properties (Le Guezennec *et al*, 2006). Proteins interacting with MBD2/Mi-2 complex, but not with MBD3/Mi-2 complex, have been identified, for instance PRMT5, an arginine methyltransferase (RMT) (Fabbrizio *et al*, 2002). This model proposes a mechanism in which methylated DNA would be recognised by MBD2/Mi-2 complex leading to arginine methylation and histone hypoacetylation of the histone next to methylated DNA. This new state of the chromatin structure would be the target of MBD3 containing Mi-2 complex enhance histone deacetylation spreading to proximal regions.

## MBD4

Methyl-CpG-binding domain 4 is part of the DNA repair machinery. MBD4 possesses the MBD at the N-terminal region and a glycosylase domain at the C-terminal region. This protein recognizes methylated CpG sequences but it has more affinity for 5mCpG-TpG sequences, a product of 5mCpG dinucleotide deamination. This deamination of 5-mC to thymine occurs spontaneously and MBD4 triggers DNA repair at this points by interacting with MLH1 (mutL homologue 1) (Bellacosa *et al*, 1999). MBD4 might also have an essential role in the triggering of apoptosis upon DNA damage (Zabkiewicz and Clarke, 2004).

MBD4 knock out mice show more cytosine to thymine transitions at CpG sites (Millar *et al*, 2002). Moreover, MBD4^−/−^ mice with a Apc^(Min/+)^ background develop more intestinal tumours than Apc^(Min/+)^ MBD4^+/+^ mice. The correlation between MBD4 deficiency and intestinal tumorigenesis has also been assessed in humans, in which 26–43% of microsatellite unstable colorectal tumours show mutations in the MBD4 gene (Riccio *et al*, 1999). Thus, MBD4 might act as a tumour suppressor gene in human malignancies.

Although MBD4 is mainly related to DNA repair mechanism, it is also involved in methylation-dependent transcriptional repression of p16^INK4a^ and MLH1 genes (Kondo *et al*, 2005).

## OTHER METHYLATED CPG-BINDING PROTEINS

Other proteins with the ability to selectively bind methylated DNA have been described. The archetypical member of this family of proteins is Kaiso, a member of the BTB/POZ (POZ/ZF) zinc finger domain family of proteins that binds methylated CpGs in the context of a consensus sequence composed by at least two consecutive methylated CpG dinucleotides. Repressive activity of Kaiso is mediated by N-CoR corepressor complex, which includes HDAC3 (Klose and Bird, 2006). Kaiso also recognises the consensus sequence TCCTGCNA (N being any nucleotide) throughout a methylated DNA-binding domain independent region. The affinity for this sequence is higher that the affinity for methylated CpGs and it could be involved in specific DNA sequences binding (Daniel *et al*, 2002). Kaiso knockout mice intercrossed with Apc^(Min/+)^ background are less susceptible to intestinal tumours (Prokhortchouk *et al*, 2002) than Apc^(Min/+)^ kaiso^+/+^ mice.

Two other proteins with kaiso like zinc fingers were also described, ZBTB4 and ZBTB38. Both proteins bind methylated DNA and it seems that a unique methylated CpG dinucleotide is sufficient for their binding and gene silencing induction (Filion *et al*, 2006).

## TARGETING MBD PROTEINS

Methyl-CpG-binding domain proteins are being used in translational approaches to identify new hypermethylated genes in human cancer. Three representative examples are the use of antibodies against MBDs in association with chromatin immunoprecipitation and genomic arrays (Ballestar *et al*, 2003), the association of an MBD to a column that allow the capture of methylated sequences from stools of patients with colorectal cancer (Zhou *et al*, 2007) and the depletion of MBDs to release the transcriptional silencing of putative tumour suppressor genes (Lopez-Serra *et al*, 2008).

Most important, DNA methylation and elements of the epigenetic machinery constitute very interesting targets for the design of therapeutic compounds (Esteller 2008). Actually, the use of demethylating drugs such as 5-azacytidine and 5-aza-2′-deoxycytidine (base analogs that avoid DNMTs function) has been approved for cancer therapy, specifically for the treatment of myelodysplastic syndrome and acute myeloid leukaemia. The unspecific reactivation of methylated sequences is the main problem of epigenetic treatments. Parallel with demethylation of tumour suppressor genes CpG islands, global genomic demethylation, which could cause or contribute to chromosomal instability, also occurs. Instead of targeting DNA methyltransferases, the proteins that recognise the signal encoded by DNA methylation and recruit histone modifying and chromosome remodelling complexes to these methylated sequences, such as MBDs, could also be good targets in cancer therapy. This strategy could avoid problems associated with genomic instability which inhibiting DNA methylation *per se* could induce. It is necessary to determine specific targets of each MBD protein and the exact role they play in cancer progression to specifically target those involved in each tumour type. Thus, in a similar manner for the targeted therapy against gene mutations, there are prospects for the development of directed epigenetic specific therapy, using for example designed transcription factors that target particular gene promoters and are able to re-activate the MBD-silenced gene.

## Figures and Tables

**Table 1:** MBD proteins bound to promoter hypermethylated CpG islands of tumour suppressor genes

	**Target genes**
MeCP2	RASSF1A, RARB2, GSTP1, MGMT, CDH1, CDH13, LTBP3, PARVG, COLL11A2
MBD1	GSTP1, SOCS-1, COL11A2, PTPRN, FGF19, RARB2, CDH1
MBD2	BRCA1, MGMT, p16^INK4a^, p14ARF, LTBP3, PTPRN, ER1, TIMP3, CDH1, GSTP1, PRLR, PTPN4
MBD3	P21^*^, CHFR, PTPN4
MBD4	p16^INK4a^, MHL1

p21^*^ unmethylated CpG island.

References for the target genes are included in the text.
